# Integrated Needle Guide on Smartphone for Percutaneous Interventions Using Augmented Reality

**DOI:** 10.1007/s00270-025-04044-4

**Published:** 2025-04-28

**Authors:** Laetitia Saccenti, Nicole Varble, Tabea Borde, Hannah Huth, Michael Kassin, Ifechi Ukeh, Ivan Bakhutashvili, Ashley Golbus, Lindsey Hazen, Sheng Xu, Vania Tacher, Hicham Kobeiter, Keith Horton, Ming Li, Bradford Wood

**Affiliations:** 1 Center for Interventional Oncology, Radiology and Imaging Sciences, Clinical Center, National Institutes of Health, 10 Center Drive, Bethesda, MD 20892 USA; 2 Henri Mondor’s Institute of Biomedical Research - Inserm, U955 Team N°18, Creteil, France; 3 Philips Research of North America, Cambridge, MA USA; 4 Department of Radiology, Henri Mondor University Hospital, Assistance Publique - Hopitaux de Paris (AP-HP), Creteil, France

**Keywords:** Augmented reality, Image-guided therapy, Percutaneous procedure, Interventional radiology

## Abstract

**Purpose:**

This study aimed to describe the workflow and evaluate the accuracy of a novel smartphone augmented reality (AR) application that includes an integrated needle guide, in a phantom.

**Materials and Methods:**

A smartphone cover with an integrated needle guide was designed and 3D-printed. An AR application for percutaneous application was developed, which integrated a projected needle path based on the rigid needle guide. After planning the needle path using this novel tool, the operator could place the needle through the guide to reach the target. Six lesions with out-of-plane entry points were targeted on an abdominal phantom. Timing and accuracy of needle placements were measured on post-procedural CT both using smartphone AR guidance and with a freehand approach. Results were compared using Wilcoxon rank sum and Pearson’s chi-squared tests.

**Results:**

A total of 108 needle placements were performed by 9 physicians with widely varying experience. The median accuracy was 4 mm (IQR 3-6 mm) using the smartphone versus 18 mm (IQR 9-27 mm) for freehand (*P* < 0.001). Using the smartphone AR application, planning time was 91 s (IQR 71-151 s), and puncture time was 68 s (IQR 57-77 s). There was no difference in accuracy, planning, or puncture times according to experience level when using the AR tool.

**Conclusion:**

This smartphone application with an integrated needle guide allows path planning and accurate needle placement on phantoms with real-time angular feedback in less than 3 min. This technology could promote standardization, reduce experience requirements, or provide accurate low-cost guidance in environments without procedural imaging for percutaneous interventions.

**Graphical Abstract:**

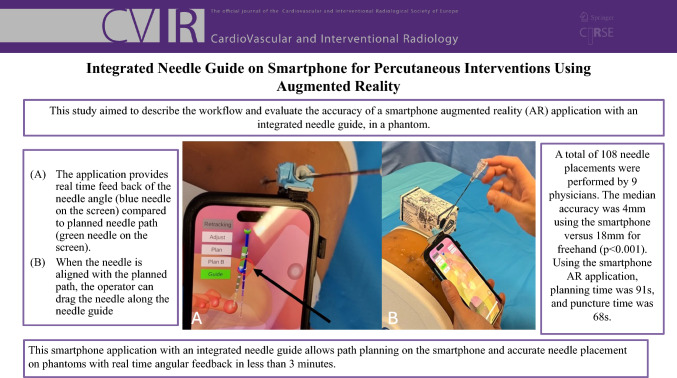

**Supplementary Information:**

The online version contains supplementary material available at 10.1007/s00270-025-04044-4.

## Introduction

Augmented reality (AR) overlays digital information or virtual objects onto the real world. In the context of medicine, AR can be used to superimpose medical scans or clinical information onto the patient or the operator’s field of view. As a result, this technology can enhance visualization during surgical or image-guided procedures [1–3], medical training [4–6], and patient education [7].

The combination of percutaneous intervention and augmented reality has the potential to standardize and propagate minimally invasive medical procedures [8–11]. Specifically, AR can assist physicians in accurately navigating needles and catheters [12] to precise locations within the body, providing real-time feedback and enhancing procedural safety and efficacy [13, 14], regardless of the operator experience [15], and potentially without requirements for conventional intra-procedural imaging. When applied to the field of interventional radiology, AR can serve to improve the ergonomics of the intervention [16] or improve treatment planning by providing a spatial iterative record for composite ablations [17].

AR applications in the medical field may be implemented through the use of a goggle headset [18], to ensure that operators can maintain a hands-free interaction. While seemingly ideal for intraoperative applications, headsets also come with some limitations including cost, discomfort, eye strain, fatigue, and cybersickness [19, 20]. Smartphones are ubiquitous and are an accessible and familiar hardware for AR experiences [21, 22]. Given that users do not need to invest in specialized AR devices, smartphone AR is a more cost-effective and accessible option for experiencing and utilizing AR [23]. Moreover, smartphones offer a seamless integration of AR with other device features, such as cameras, gyroscopes, inertial measurement unit (IMU), and touchscreens [24, 25]. This integration enables richer and more interactive AR experiences that leverage and rechannel multiple sensors and functionalities of the smartphone.

Smartphone AR applications do, however, face some disadvantages, namely ergonomics of use. Smartphone handling, setup, and workflow is learned and may not be intuitive. Some have found solutions to overcome such barriers including mounting the device on a flexible arm [26] or a dedicated stand [28], or having a colleague to hold it [27]. Another proposed solution was to touch left and right hands together to provide stabilization for needle in one hand and smartphone in the other [14]. In this study, a needle holder was fixed on the smartphone cover with custom software, to improve the ergonomics of the smartphone AR guidance for percutaneous interventions.

This study aimed to describe the workflow and evaluate across experience levels the accuracy of an AR application for a smartphone with an integrated needle guide, in a phantom.

## Material and Methods

### Device Overview: Smartphone Cover with Needle Holder for AR Application

**Fig. 1:**
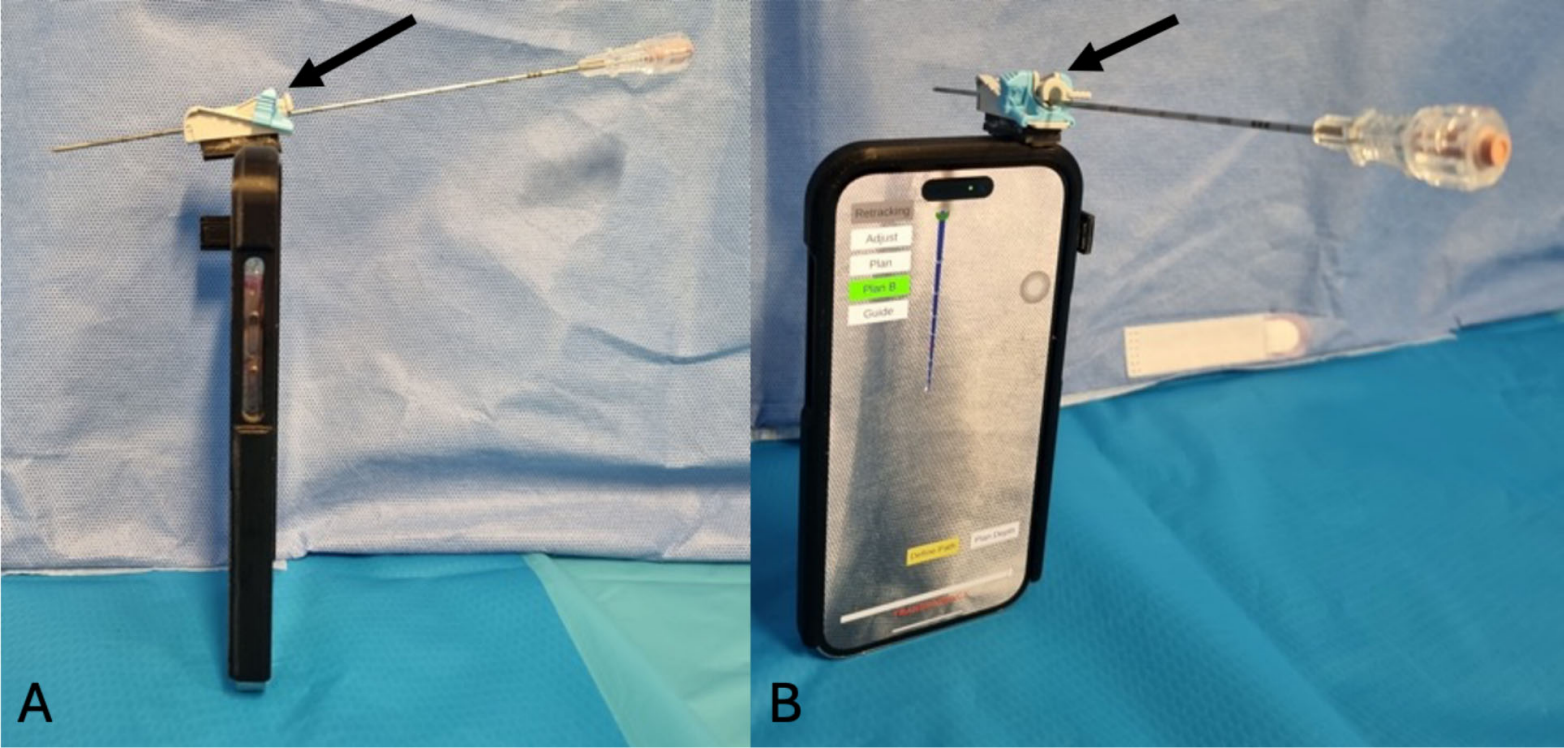
Lateral **A** and oblique **B** views of the smartphone cover with integrated needle guide (arrow). The smartphone cover was designed and 3D-printed to adapt a Verza needle guide (Civco) on its top side

A smartphone cover for iPhone 14 Pro with an integrated needle guide (Verza needle guide, Civco) was designed and 3D-printed (UltiMaker S3, Ultimaker B.V., the Netherlands) (Fig. 1). This needle guide design was chosen because of its ability to hold different sizes of needles, modify the needle angle, and easily detach the needle from the guide hardware after placement. The needle guide can be attached to the smartphone cover (like a guide attaches to a covered ultrasound), and the guide is sterile, FDA-cleared, and disposable.

An AR application for percutaneous interventions was developed on Unity. The fixed needle guide enables the projected needle path to be implemented into the application. The AR application used a smartphone camera, embedded gyroscope, and IMU sensors to register the preoperative CT with the target body and track the phone location relative to the patient or phantom in real time. This enabled the operator to plan the needle path at the bedside and provided needle alignment feedback from multiple viewing angles, using both visual and gyroscopic confirmation.

### Phantom Experiment

An anthropomorphic abdominal phantom (Model 057A, Sun Nuclear, Melbourne, FL) with preexisting target lesions was used.

Operators of varying levels of expertise were recruited to perform smartphone AR-assisted punctures followed by freehand punctures, using a 17G needle. Six targets (liver: *n* = 5, kidney *n* = 1) were designated in a random order and shuffled for each operator. The median diameter of the target was 9 mm (range 7–13 mm). The entry points were pre-defined for variable complexity, to have only out-of-plane paths, with various insertion angles. The median length of the needle paths was 82 mm (range 52–99 mm).

### Smartphone AR Guidance

**Fig. 2:**
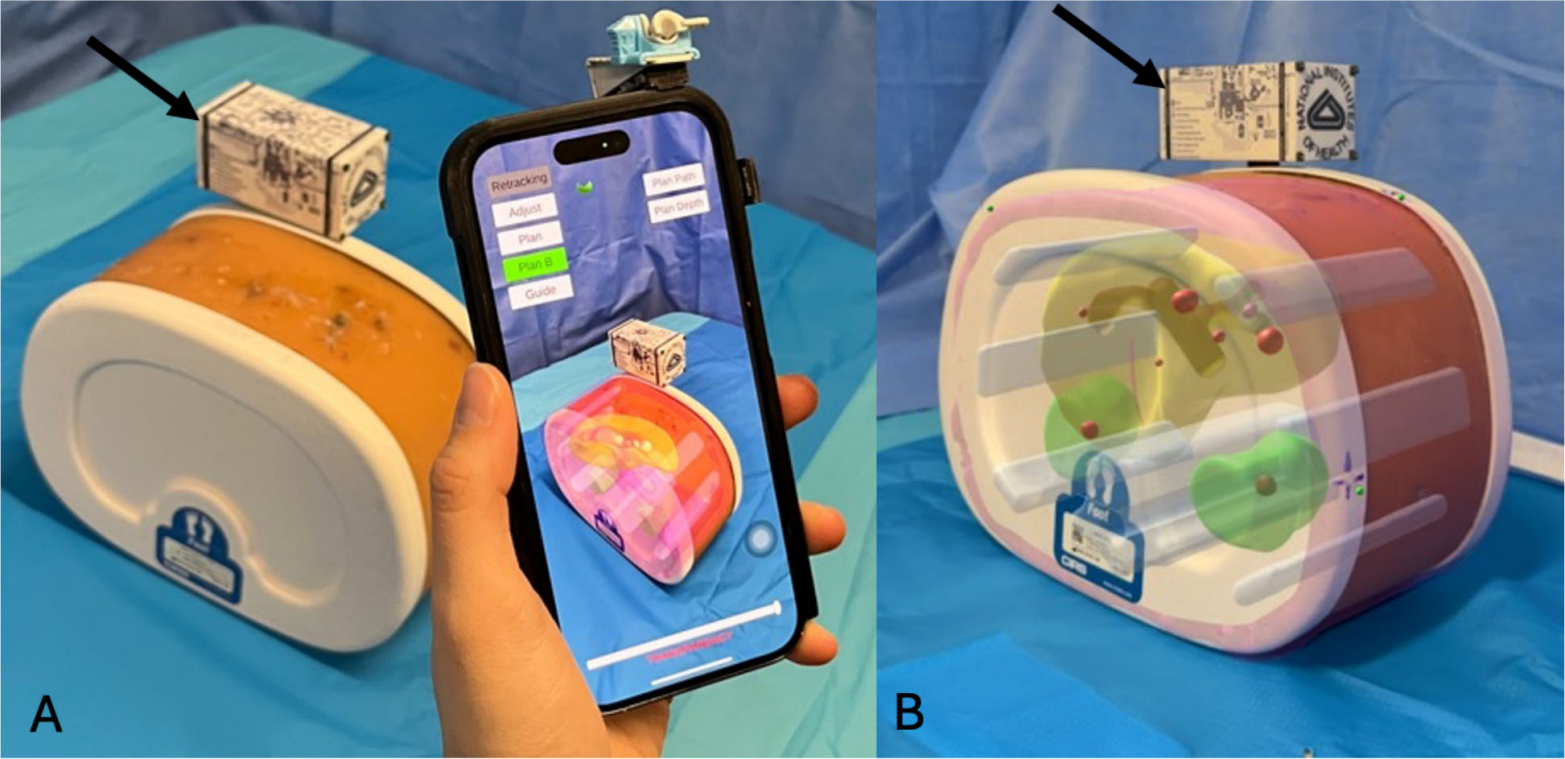
Registration of the 3D model on the phantom via video of a fiducial reference box (arrow). **A** View of the operator. **B** View of the smartphone screen

Prior to the procedure, the abdominal phantom CT was segmented and modeled using an open-source software (3D slicer, https://www.slicer.org/). Preprocedural segmentation was not included in the planning time. CT (slice thickness: 0.8 mm, voxel size: 512 × 512 × 467) (Philips IQon Spectral CT, Best, The Netherlands) was displayed and projected on the smartphone through the AR application. Registration of the 3D model on the phantom was made using a fiducial box included in the preprocedural CT (Fig. 2), as previously described [28]. Prior to the experiment, all operators had a training session that consisted of 1 to 4 needle placement using AR guidance, until the operator felt confident.

### Smartphone AR Workflow

**Fig. 3:**
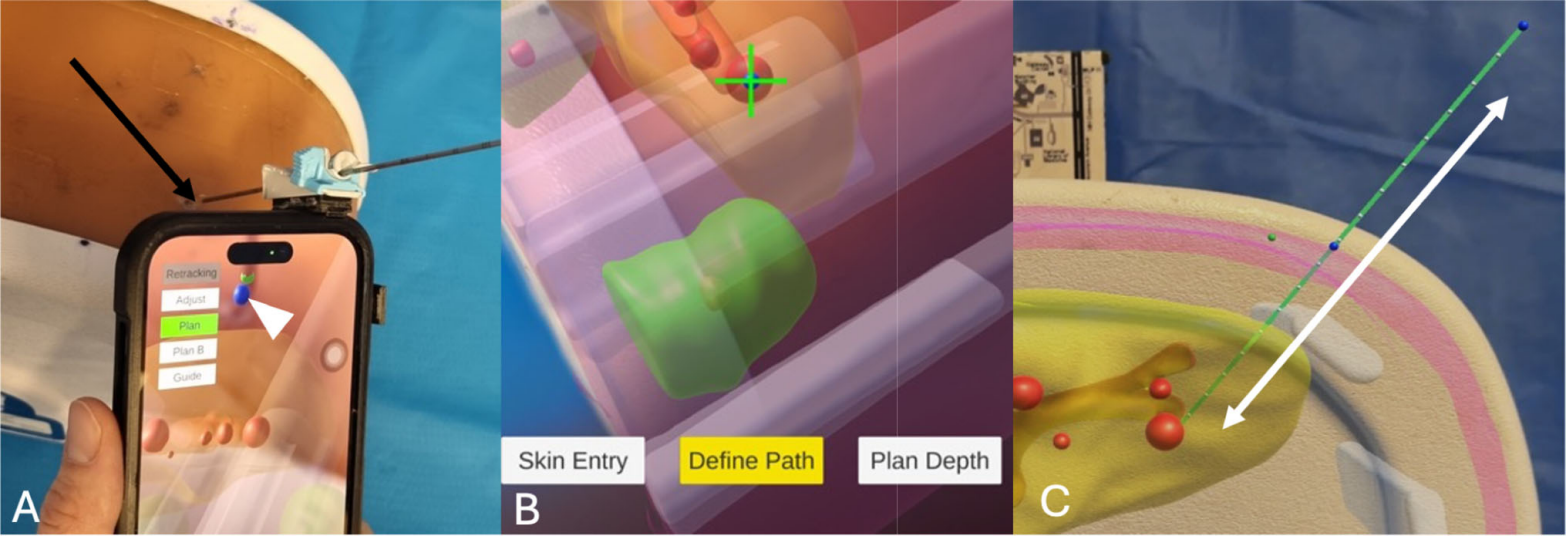
Workflow: planning the needle path. **A** The location of the entry point in the augmented reality space is recorded (white arrowhead) by touching it with the tip of the real needle (black arrow). **B** View from the smartphone application. The planned path of the needle is defined by aligning the entry point (blue dot) with the target (red sphere), at the center of the screen (green cross). **C** View from the smartphone application. The planned needle path had the exact length of the needle. The depth of the planned needle path was adjusted on the smartphone screen by sliding it (white arrow) until it reaches the target

Planning needle path: A fixed entry point for the needle was selected by the operator and recorded in AR-space by touching the position with the tip of the needle (Fig. 3A). Then, the operator defined the needle path by aligning the entry point with the target on the screen (Fig. 3B). The needle path length was pre-defined as the length of the physical needle by the application. The needle path depth was adjusted using a slider bar to position the virtual needle on its planned trajectory until the distal extremity reached the target (Fig. 3C). Therefore, the virtual needle as a planned path was overlaid onto the phantom.

**Fig. 4:**
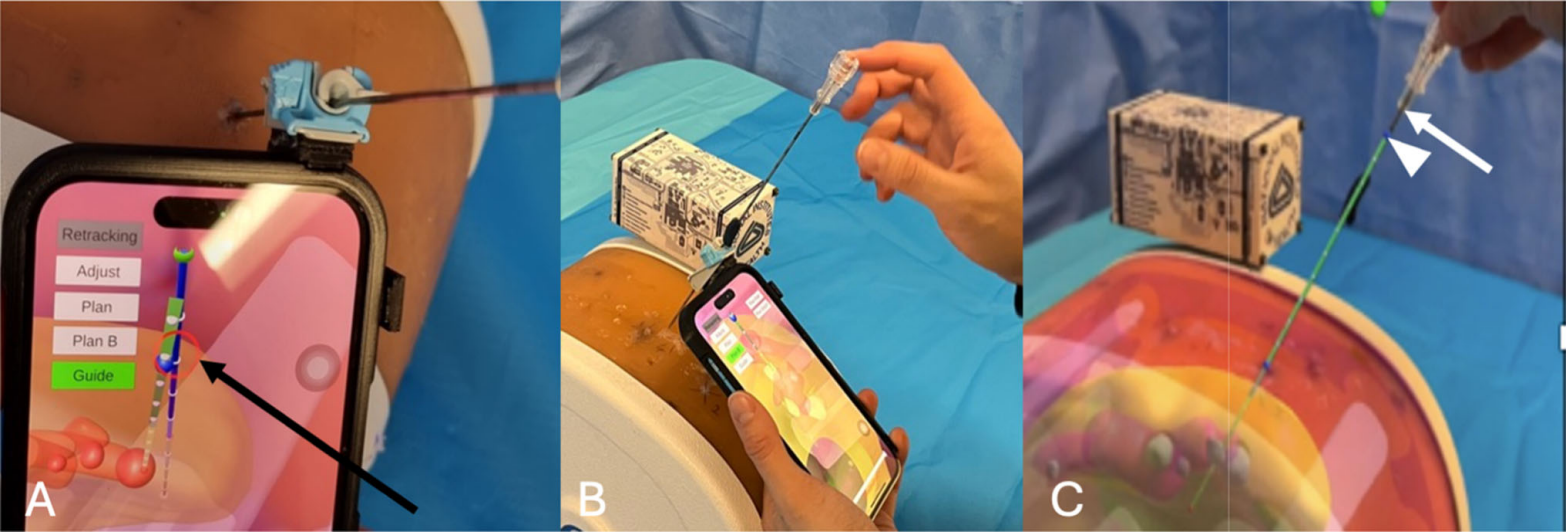
Workflow: needle guidance. **A** View of the operator. The application provides real-time feedback of the needle angle (blue needle on the screen) compared to planned needle path (green needle on the screen). When both are aligned, the red circle (arrow) becomes smaller and green. **B** View of the operator. When the needle is aligned with the planned path, the operator can drag the needle along the needle guide. **C** View through the smartphone. After detaching the needle from the needle guide, the operator can adjust the depth of the needle by matching the hub–needle interface of the needle (white arrow) with the planned path (white arrowhead)

Needle guidance: The operator placed the tip of the needle on the entry point and aligned the needle with the planned needle path, with the help of an angular feedback from the smartphone gyroscope (Fig. 4A). While the needle was aligned with the planned path, the operator inserted the needle through the guide toward the pre-defined target (Fig. 4B). After detaching the needle from the needle guide, the operator adjusted the depth of the needle by matching the hub–needle shaft interface with the virtual needle (Fig. 4C).

A video of the smartphone AR workflow is available in supplemental material.

### Freehand Guidance

The operators had access to preprocedural CT with marked entry points and multiplanar reconstruction to plan needle insertions. Interval CT imaging during needle placement was not permitted, to equalize radiation exposure between cohorts.

### Outcomes

Planning time and puncture time were recorded. Using AR guidance, planning time was the time to plan the needle path using the smartphone application. Puncture time was the time to align the needle with the planned path, insert the needle, and adjust its depth. Using freehand guidance, planning time was the time to prepare needle insertion by analyzing CT images with multiplanar reconstruction. Puncture time was the time to insert the needle in the phantom.

**Fig. 5:**
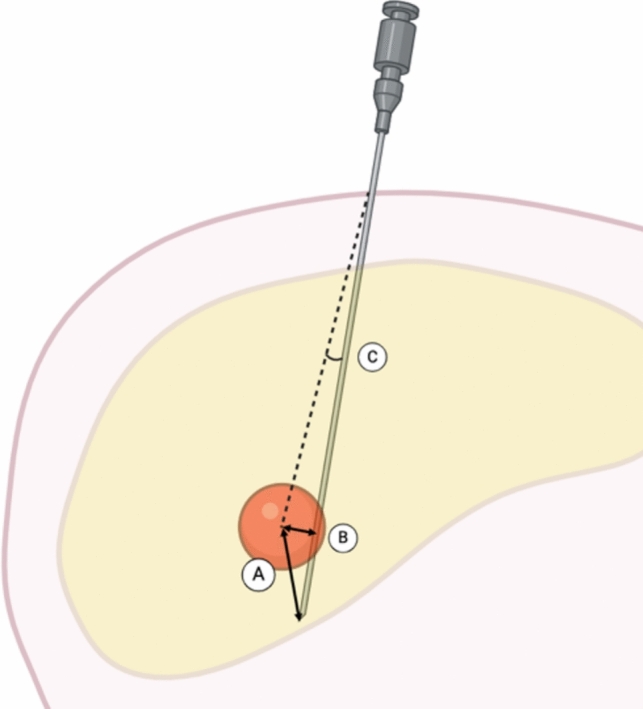
Accuracy metrics on post-procedural CT, evaluated by measuring distance between the needle tip and the center of the target (**A**), shortest distance from needle shaft to the center of the target (**B**), and angular error (**C**)

A post-procedural CT was performed after every 3 needle insertions to avoid clutter. A target was “reached” by the needle if the needle touched it. Accuracy was evaluated by measuring the Euclidean distance, from the tip of the needle to the center of the target. The angular error was also measured, and the shortest orthogonal distance from the needle shaft to the center of the target was calculated (Fig. 5).

### Statistics

The normality of data was assessed using the Shapiro–Wilk test. Results are presented as median (Interquartile range [IQR]). Continuous variables were compared using the Wilcoxon rank sum test, and categorical data were compared using Pearson’s chi-squared test. Comparisons between operator’s experience or according to each target location were performed using the Kruskal–Wallis rank sum test for continuous data and Fisher’s exact test for categorical data. A *p*-value < 0.05 was considered as statistically significant.

All statistical tests were computed in the open-source software R (R Foundation for Statistical Computing, Vienna, Austria. URL https://www.R-project.org/).

## Results

A total of 108 needle placements (54 for each guidance technique) were performed by 9 physicians with widely varying IR experience: 2 medical students, 2 residents or trainees, 3 attendings with 5–10-year experience, and 2 senior attendings with more than 20-year experience.

### Accuracy

**Fig. 6:**
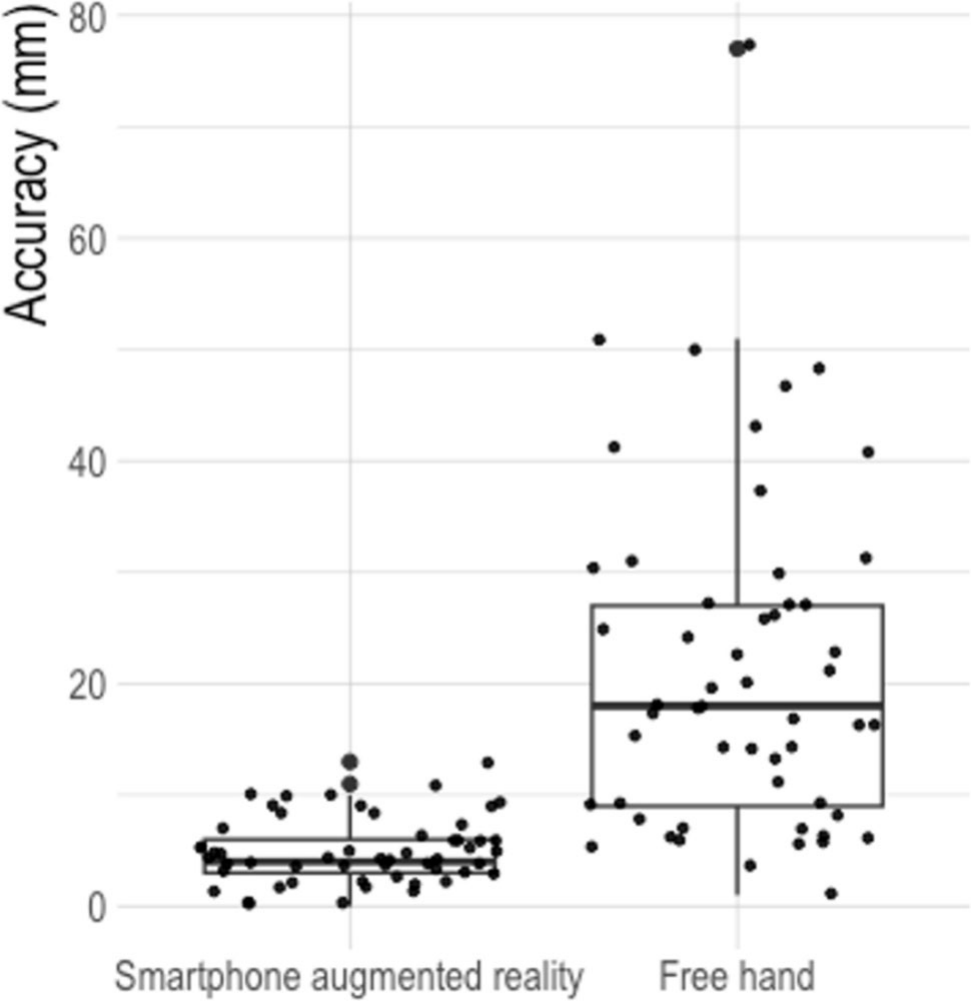
Standardization with use of the smartphone augmented reality guidance, shown with comparative accuracy of needle placement using the freehand vs. smartphone augmented reality guidance

Using the smartphone AR application, 78% (42/54) of the targets were successfully reached by the needles, versus 24% (13/54) (*P* < 0.001) using freehand. The median accuracy (from the needle tip to the center of the target) was 4 mm [IQR 3–6 mm] using the smartphone versus 18 mm [IQR 9–27 mm] for freehand (*P* < 0.001). The median angular error was 2.7° [IQR 1.6–4.6°] using the smartphone versus 9° [IQR 5.2–17.1°] for freehand (*P* < 0.001). The median distance from the needle shaft to the center of the target was 4 mm [IQR 2–5 mm] using the smartphone versus 13 mm [IQR 7–22 mm] for freehand (*P* < 0.001). Results are detailed in Table 1 and Fig. 6.

**Table 1:** Accuracy of needle placements in a phantom using smartphone augmented reality guidance compared to freehand

	Smartphone augmented reality (N = 54)	Free hand (N = 54)	*P*-value^3^
Reached targets^1^	42 (78%)	13 (24%)	< 0.001*
Distance (mm) from needle tip to target center^2^	4 [3–6]	18 [9–27]	< 0.001*
Distance (mm) from needle shaft to target center^2^	4 [2–5]	13 [7–22]	< 0.001*
Angular error (°)	2.7 [1.6–4.6]	9.0 [5.2–17.1]	< 0.001*

*Statistically significant

^1^N(%)

^2^Median [Interquartile range]

^3^Pearson’s chi-square test/Wilcoxon rank sum test

### Timing

Using the smartphone AR application, planning time was 91 s [IQR 71–151 s], and puncture time was 68 s [IQR 57-77 s]. Both were slightly longer than freehand placement, which had planning time of 73 s ([IQR46-115 s], *P* = 0.01) and puncture time of 31 s ([IQR 20–46 s], *P* < 0.001).

### Smartphone AR Learning Curve

When comparing the first set of 3 randomized needle placements to the second set of 3 randomized needle placements, using the smartphone AR application, no difference was found in terms of planning time (first set: 104 s [IQR 77–147 s] versus second set 87 s [IQR 71–164 s], *P* = 0.80), puncture time (first set: 72 s [IQR 57–88 s] versus second set: 67 s [IQR 57–73 s], *P* = 0.24), or accuracy (first set: 4 mm [IQR 3–7 mm] versus second set 4 mm [IQR 4–6 mm], *P* = 0.85).

### Operator’s Experience

Using smartphone AR guidance, no difference was found according to operator’s experience in terms of accuracy (*P* = 0.81), planning time (*P* = 0.69), or puncture time (*P* = 0.06, Table 2).

**Table 2:** Performances of smartphone augmented reality guidance on a phantom according to operator’s experience

	Medical students (N = 12)	Resident/trainees (N = 12)	Attendings (N = 18)	Senior attendings (N = 12)	*P*-value^4^
Planning time (s)^1^	78 [61–125]	103 [85–124]	97 [63–161]	88 [79–162]	0.69
Puncture time (s)^1^	60 [46–75]	62 [43–71]	72 [60–83]	82 [59–113]	0.06
Reached target^2^	10 (83%)	8 (67%)	16 (89%)	8 (67%)	0.36
Accuracy (mm)^1,3^	5 [2–9]	4 [3–7]	4 [4, 5]	5 [3–6]	0.81

^1^Median [Interquartile range]

^2^N(%)

^3^Distance from needle tip to target center

^4^Kruskal–Wallis rank sum test; Fisher’s exact test

Using freehand, no difference was found according to operator’s experience in terms of accuracy (*P* = 0.20), planning time (*P* = 0.22), or puncture time (*P* = 0.31).

### Target Location

Using smartphone AR guidance, differences in terms of accuracy (*P* = 0.024) and angle error (*P* < 0.001) were found according to the target location. Using freehand guidance, no differences in terms of accuracy (*P* = 0.37) or angle error (*P* = 0.63) were found according to target location (Table 3). The smartphone application error was less accurate in specific pathways than others, compared to freehand, which varied regardless of obliquity.

**Table 3:** Performances of needle insertions using smartphone augmented reality guidance and free hand on a phantom according to target location

Target number								
		1, N = 9^*1*^	2, N = 9^*1*^	3, N = 9^*1*^	4, N = 9^*1*^	5, N = 9^*1*^	6, N = 9^*1*^	*P*-value^*2*^
Smartphone augmented reality	Angular error (°)	4.7 [4.5- 6.1]	0.4 [0.0- 0.9]	3.5 [2.1- 4.5]	1.8 [1.4- 2.5]	3.2 [2.5- 3.5]	3.3 [2.8- 5.2]	< 0.001*
	Accuracy (mm)	5 [4–9]	2 [0- 4]	5 [3–8]	5 [3–5]	6 [4–9]	4 [4–6]	0.024*
Free hand	Angular error (°)	8.6 [5.6- 19.3]	8.0 [2.7- 26.5]	15.2 [9.2- 16.5]	11.8 [5.1- 17.3]	10.7 [5.5- 14.3]	6.4 [5.6- 8.8]	0.63
	Accuracy (mm)	8 [6–27]	16 [7- 48]	18 [16–25]	27 [14- 41]	23 [18–26]	9 [8–17]	0.37

*Statistically significant

^1^Median [Interquartile range]

^2^Kruskal–Wallis rank sum test

## Discussion

The integration of a needle guide to a smartphone AR application allowed needle path planning (target and entry point selections) directly on the application with fixed geometry on needle phone guide. The addition of a needle guide theoretically simplifies the complexities required of the operator in estimating the relationship of needle to phone camera, in that it reduces the unrestricted nature of the degrees of freedom. With the needle guide, the smartphone is used to give the operator real-time feedback. In this constrained phantom study, smartphone AR accuracy outperformed freehand insertion.

Previous studies have shown the potential of smartphone AR applications to guide percutaneous interventions. Smartphone AR applications have been described to project needle paths [14, 17, 28, 29] or give angular information [21, 23, 24, 30], while the operator must manually adjust the needle, giving real-time visual feedback to the operator, thanks to the smartphone camera. This may involve holding the smartphone in one hand and the needle in the other hand (or hands touching), which requires spatial coordination skills to stabilize both devices. Moreover, planning the needle trajectory had to be pre-planned on a workstation prior to the procedure [14, 17, 29]. While integrating a needle guide fixed on the smartphone, the operator can hold both the needle and smartphone at the same time. The fixed path acts as a connector from the real world into the AR world, enabling new possibilities and workflows with the AR application. The smartphone needle guide component allows planning the path on the smartphone application, at the patient’s bedside, via the smartphone’s IMU and gyroscope to track the position and orientation of the needle. The novel smartphone iteration provides the operator more robust “sensors-based” real-time feedback. In this phantom study, planning the path on the smartphone application was feasible and rapid, with a median time of 91 s, while the insertion itself took 68 s.

In this abdominal phantom study, the accuracy (median distance from the needle tip to the center of the target) using smartphone AR guidance was 4 mm, which was similar to the distance from the needle shaft to the center of the target. Therefore, depth and angle error both contributed to the overall error. The smartphone accuracy far outperformed freehand accuracy, which was 18 mm. Only out-of-plane needle paths were chosen, including a lateral entry point to reach a kidney target, but no difference by target location was found in terms of accuracy using freehand. Interestingly, some targets were more difficult to reach using the smartphone AR guidance, with significant differences in accuracy (*P* = 0.024) and angular error (*P* < 0.001). Those inaccuracies suggest the position of the smartphone compared to the needle path could have some impact on the precision and accuracy of the guidance. Indeed, some sensors, like the smartphone gyroscope, may work better when holding the phone in a vertical position to gravity. However, the smartphone AR accuracy was similar to previously published accuracy ranges in phantoms using both goggle-based AR guidance: 3.6–5.2 mm [14, 15, 29, 31], and smartphone-based AR guidance: 2.6–4 mm [14, 29], while the other devices did not include path planning in the workflow. Table 4 compares the differences of workflow and potential sources of error between the smartphone AR with integrated needle-guide presented in this study and the previous version without needle guide previously tested [14, 28, 29].

**Table 4:** Comparison of workflow and potential sources of error between the smartphone augmented reality application with integrated needle guide compared to the previous base version without needle guide

Technology	Previous base version of the smartphone AR application without needle guide[14, 28, 29] The operator uses the AR path (green line on the smartphone) as visual reference. Picture reproduced with permission, from: *Cardiovasc Intervent Radiol* 43, 756–764, https://doi.org/10.1007/s00270-019-02403-6 (2020)	Smartphone augmented reality application with integrated needle guide The operator uses the smartphone with needle guide to align the needle (blue path) with the planned path (green path). When both match, the angular feedback (arrow) becomes small and green
Target	Target selection prior to use on a workstation to define ideal path. Prior reports had pre-selected targets	Target selection on unified smartphone application itself. Ideal path planning is all on one unified smartphone app workflow. This experimental plan includes error introduced by target selection (Potential for error is not inherent to technology)
Entry point	Entry point selection prior to use on a workstation. A radiopaque surface grid is required and included in preprocedural CT prior reports had pre-determined targets	Entry point selection is based on AR, and one unified smartphone app. Entry point recorded by the operator by touching the surface of the phantom with the needle tip. Error includes AR registration
Needle	Needle insertion depends upon visual assessment from multiple different angles of view	Needle insertion restricted to pre-defined guide with angular feedback from the smartphone. Error less susceptible to multiple simultaneous angulation uncertainties

No difference was found using smartphone AR guidance between the first and the second set of 3 needle placements, in terms of planning time (*P* = 0.80), puncture time (*P* = 0.24), or accuracy (*P* = 0.85). Therefore, no training effect was detected after the first set of punctures, implying the short training provided before the experiment (1 to 3 needle placements, until the operator felt confident) was sufficient. Finally, no difference was found according to operator’s experience in interventional radiology, confirming the potential of AR to promote standardization, reduce the influence of operators’ experience [15], and potentially enable less experienced to gain confidence for specific needle-based tasks. The smartphone with needle guide might facilitate training and help trainees assess their skillsets in a rapid reproducible fashion without the time required for iterative CT confirmation.

This study presents several limitations: The accuracy has been measured on a phantom, which allows reliable registration thanks to the fiducial box, without breathing movement, and without modification of the anatomy and of the fiducial position between preprocedural CT and needle placement. Respiratory motion was not addressed in this model. Beyond the scope of this initial study might be a comparison of predictive gating models, motion correction, deformable registration, and dynamic deformable registration, which are shared concerns for any navigation methodology. Then, the accuracy of the smartphone AR guidance was compared to freehand guidance without intermediate CT controls, to equalize radiation exposure among cohorts. The needle paths were pre-defined with double obliquity and wide angles, which made them intentionally challenging to reach using free hand, even for experienced operators. In actual clinical workflow, intermediate CT controls, or ultrasound, CBCT, or CT fluoroscopy or step and shoot CT techniques might have been used. The impact or possibility of whether this new approach could be combined with conventional image guidance tools was not studied. It is hard to reproduce past conclusions whereby less experienced operators were improved more than experienced, since both groups had improved performance. Finally, the application required the upload and segmentation of the 3D model of the phantom from preprocedural CT images, which can be time-consuming, without semiautomatic algorithms. Hopefully deep learning-based segmentation software will help with this task in the near future. However, further refinements and research and development are still needed to fully investigate the potential impact of smartphone AR technology in clinical practice.

## Conclusion

This smartphone application with an integrated needle guide is a novel low-resource guidance tool that allows path planning on the smartphone and accurate needle placement (4 mm error on phantoms) with real-time angular feedback in less than 3 min, regardless of operator experience. The addition of a needle guide theoretically simplifies the complexities required of the operator in estimating the relationship of needle to phone camera, in that it reduces the unrestricted nature of the degrees of freedom. This technology does not require extensive training, may reduce the influence of experience on accuracy of percutaneous interventions, and provides a novel way to unite the mixed reality world with IR without goggles.

## Supplementary Information

Below is the link to the electronic supplementary material.

Supplementary file1 Video 1: AR smartphone workflow.
